# Diagnostic accuracy of artificial intelligence in detecting left ventricular hypertrophy by electrocardiograph: a systematic review and meta-analysis

**DOI:** 10.1038/s41598-024-66247-y

**Published:** 2024-07-10

**Authors:** Noppachai Siranart, Natee Deepan, Witina Techasatian, Somkiat Phutinart, Walit Sowalertrat, Ponthakorn Kaewkanha, Patavee Pajareya, Nithi Tokavanich, Narut Prasitlumkum, Ronpichai Chokesuwattanaskul

**Affiliations:** 1 Division of Cardiology, Department of Medicine, Faculty of Medicine, Chulalongkorn University and King Chulalongkorn Memorial Hospital, Thai Red Cross Society, 1873 Rama 4 Road, Pathumwan, Bangkok, 10330 Thailand; 2 Division of Cardiovascular Medicine, Center of Excellence in Arrhythmia Research, Cardiac Center, King Chulalongkorn Memorial Hospital, Faculty of Medicine, Chulalongkorn University, Bangkok, Thailand; 3 Department of Biochemistry, Faculty of Medicine, Chulalongkorn University, Bangkok, 10330 Thailand; 4 Department of Medicine, John A. Burns School of Medicine, University of Hawai’i, Honolulu, HI USA; 5 Division of Cardiovascular Medicine, Frankel Cardiovascular Center, University of Michigan Health, Ann Arbor, MI USA; 6 Department of Cardiovascular Medicine, Mayo Clinic College of Medicine, Rochester, MN USA

**Keywords:** Artificial intelligence, Left ventricular hypertrophy, Electrocardiogram, Accuracy, Diagnostic tool, Cardiology, Diagnosis, Physical examination, Outcomes research

## Abstract

Several studies suggested the utility of artificial intelligence (AI) in screening left ventricular hypertrophy (LVH). We hence conducted systematic review and meta-analysis comparing diagnostic accuracy of AI to Sokolow–Lyon’s and Cornell’s criteria. Our aim was to provide a comprehensive overview of the newly developed AI tools for diagnosing LVH. We searched MEDLINE, EMBASE, and Cochrane databases for relevant studies until May 2023. Included were observational studies evaluating AI’s accuracy in LVH detection. The area under the receiver operating characteristic curves (ROC) and pooled sensitivities and specificities assessed AI’s performance against standard criteria. A total of 66,479 participants, with and without LVH, were included. Use of AI was associated with improved diagnostic accuracy with summary ROC (SROC) of 0.87. Sokolow–Lyon’s and Cornell’s criteria had lower accuracy (0.68 and 0.60). AI had sensitivity and specificity of 69% and 87%. In comparison, Sokolow–Lyon’s specificity was 92% with a sensitivity of 25%, while Cornell’s specificity was 94% with a sensitivity of 19%. This indicating its superior diagnostic accuracy of AI based algorithm in LVH detection. Our study demonstrates that AI-based methods for diagnosing LVH exhibit higher diagnostic accuracy compared to conventional criteria, with notable increases in sensitivity. These findings contribute to the validation of AI as a promising tool for LVH detection.

## Introduction

Left ventricular hypertrophy (LVH) is presently identified using a range of diagnostic techniques, such as electrocardiography (ECG), echocardiography, and cardiac magnetic resonance imaging (cMRI)^1^. Among these modalities, the utilization of ECG for LVH detection offers notable advantages in terms of time efficiency and reproducibility.

Cornell and Sokolow–Lyon criteria have been among the most widely used in defining LVH by ECG primarily relying on increased QRS voltage^2^. These criteria using summation of voltage amplitudes of S and R waves in an ECG represent the depolarization of the left ventricle which showed the electrical activity and functioning of the heart^3,4^. However, this feature is not universally discernible in all patients with LVH^5,6^ as previous investigations have revealed that the sensitivity of LVH screening ranges from 15 to 30%^7,8^ utilizing these criteria. Evidently, these conventional criteria exhibit limited efficacy owing to their insensitivity in early LVH detection, resulting in misclassification.

In contrast, the emergence of novel computational algorithms, such as deep learning and machine learning-based artificial intelligence (AI), has demonstrated remarkable performance across various medical domains, including medical imaging and diagnosis^9^. In this study, we have systematically compiled and analyzed the performance data of deep learning and machine learning-based AI algorithms in LVH detection using electrocardiography^9–16^, comparing their effectiveness with the conventional criteria. This study represents the pioneering attempt to evaluate and juxtapose the performance of AI in detecting LVH using ECG with traditional methods.

## Methods

### Literature review and search strategy

Our protocol for this meta-analysis is registered with PROSPERO (International Prospective Register of Systematic Reviews; no. CRD 42023434193). To identify studies evaluating the diagnostic accuracy of AI in detecting LVH, a systematic literature search was conducted. The search included MEDLINE, EMBASE, and the Cochrane Database of Systematic Reviews from inception until May 2023. The search was carried out independently by two investigators (N.S. and N.D.) using the terms (‘artificial intelligence’ or ‘machine learning’) and (‘left ventricular’ and (‘hypertrophy’ or ‘enlargement’ or ‘dilation’)) and ‘electrocardiograph’. Only articles published in English were included. A manual search of the references cited in the included articles was also performed. The study adhered to the preferred reporting items for systematic reviews and meta-analysis (PRISMA) statements (Table 1).

**Table 1:** Individuals’ baseline characteristics of included studies.

Authors	F De la Garza-Salazar^11^		T Kokubo^10^		Kwon^9^		Liu^12^		Liu^16^		Sparapani^13^		Zhao^14^		Khurshid^15^	
Year	2020		2022		2020		2022		2023		2018		2022		2021	
Country	Mexico		Tokyo		Korea		Taiwan		Taiwan		USA		China		England, Wales, Scotland	
Study design	Case–control study		Retrospective cohort study		Retrospective cohort study		Retrospective cohort study		Retrospective cohort study		Prospective cohort study		Retrospective cohort study		Prospective cohort study	
Population	Patients underwent an echocardiogram and an electrocardiography		Patients underwent electrocardiography within 4 weeks of the transthoracic echocardiography		Patients who underwent electrocardiography and echocardiography within 4 weeks		Patients who underwent electrocardiography and echocardiography within 30 days		Patients underwent an echocardiogram and an electrocardiography		Patients underwent a cardiac magnetic resonance imaging and an electrocardiography		Patients underwent an echocardiogram and an electrocardiography		Patients underwent a cardiac magnetic resonance imaging and an electrocardiography	
Diagnostics indicate LVH	Echocardiogram: Two-dimensional linear method		Echocardiogram: Two-dimensional linear method		Echocardiogram: Two-dimensional linear method		Echocardiogram: Two-dimensional linear method		Echocardiogram: Two-dimensional linear method		cMRI		Echocardiogram: Two-dimensional linear method		cMRI	
Defined LVH by	Left ventricular mass index (LVMI)		Left ventricular mass index (LVMI)		Left ventricular mass index (LVMI)		Left ventricular mass index (LVMI)		Left ventricular mass index (LVMI)		Left ventricular mass (LVM)		Left ventricular mass index (LVMI)		Left ventricular mass (LVM)	
Cut off of LVMI	Male 115 g/m^2^ Female 95 g/m^2^		Male 101 g/m^2^ Female 85 g/m^2^		Male 132 g/m^2^ Female 109 g/m^2^		Male 132 g/m^2^ Female 109 g/m^2^		Male 115 g/m^2^ Female 95 g/m^2^		The sex-specific 95th percentile of normalized LVM		Male 115 g/m^2^ Female 95 g/m^2^		The sex-specific 90th percentile of normalized LVM	
AI methods	C5.0 algorithm		ENN		ENN		CNN		BPN		BART		CNN		CNN	
Accuracy	0.714		0.732		0.88		^‡^		0.961		^‡^		^‡^		^‡^	
Sensitivity	0.796		0.605		0.454		0.903		0.966		0.29		0.68		0.34	
Specificity	0.53		0.813		0.951		0.693		0.956		0.946		0.57		0.96	
Subgroup	LVH	Non LVH	LVH	Non LVH	LVH	Non LVH	LVH	Non LVH	LVH	Non LVH	LVH	Non LVH	LVH	Non LVH	LVH	Non LVH
Total number (N)	202	230	1249	6109	4353	16,933	2435	26,310	173	779	69	871	932	931	4777	126
Mean age (years)	69.3 ± 12.1	65.7 ± 14.8	63.31 ± 17.07		67.61 ± 12.46	57.39 ± 14.89	48.6 ± 8.8	44 ± 11.3	^‡^		61.0 ± 9.9		67.3 ± 10.5	63.9 ± 11.3	63.6 ± 7.7	55.5 ± 14.6
Male (%)	55.6%		16.3%		33.2%	53.6%	52.2%	44.7%	89.9%		46.6%		45.2%	70.7%	47.5%	54%
QUADAS score	12		13		12		13		13		13		12		13	

^‡^N/A.

### Selection criteria

The eligible studies for inclusion in the review were cross-sectional, case–control, or cohort studies that assessed the diagnostic accuracy of AI, and conventional 12-lead ECG, mainly Sokolow–Lyon and Cornell criteria in detecting LVH. The articles had to provide effect estimates of overall diagnostic accuracy, sensitivity (%), and specificity (%), along with 95% confidence intervals (CIs). We selected the best AI feature defined as the highest value of area under the ROC curve from each study for further analysis. There were no limitations on the size of the studies. The two investigators independently assessed the retrieved articles for eligibility, and any discrepancies were resolved through mutual consensus. The quality of the studies was appraised using the QUADAS (Quality Assessment of Diagnostic Accuracy Studies) tool (Table 1)^17^.

### Data abstraction

A structured data collection form was used to extract the following information from each study: title, year of study, name of the first author, publication year, country of study, demographic and characteristic data of subjects, measurement of exposure, devices used for identifying and diagnosing LVH, diagnostic criteria defined by individual studies for LVH, and accuracy, sensitivity, and specificity of the AI in diagnosing LVH. Two investigators (N.S. and N.D.) independently conducted the data extraction, which was subsequently cross-checked for accuracy.

### Statistical analysis

The statistical analysis was performed using R for macOS (version 3.5.3). The R package MADA was used to calculate pooled sensitivity and specificity and generate summary receiver-operating characteristic (SROC) curves.

The adjusted point estimates from each study were combined using the generic inverse variance approach of DerSimonian and Laird^18^, which assigned weights to each study based on its variance. Due to the likelihood of increased inter-observation variance, a random-effects model was used to assess the pooled sensitivity and specificity of wearable devices, and Cochran’s *Q* test and I^2^ statistics were employed to determine between-study heterogeneity. An I^2^ value of 0–25% represented insignificant heterogeneity, 26–50% indicated low heterogeneity, 51–75% suggested moderate heterogeneity, and > 75% indicated high heterogeneity.

A bivariate random-effects regression model was used for pooling sensitivity and specificity, and SROC curves were generated based on the bivariate model. An area under the receiver operating characteristic (ROC) curve between 0.9 and 1.0 was considered excellent diagnostic accuracy, 0.8–0.9 indicated a good test, 0.7–0.8 represented a fair test, and 0.6–0.7 indicated a poor test.

A Deek’s funnel plot^19^ was generated to evaluate publication bias. A statistically significant asymmetry, indicated by a P-value less than 0.10 for the slope coefficient, was considered indicative of publication bias.

## Results

**Figure 1:**
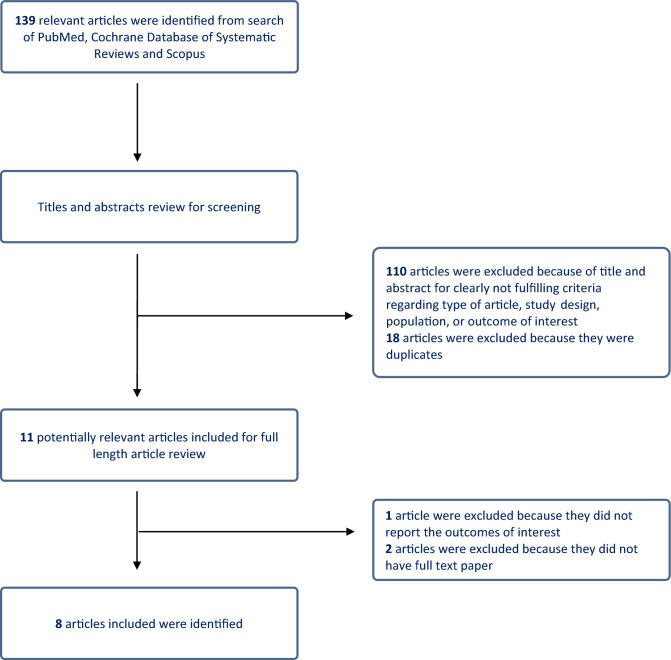
Flowchart of the literature retrieval, review, and selection processes of articles.

We initially identified 139 articles as potentially eligible through our search strategy. 18 duplicate studies were removed. After excluding 110 articles (case reports, letters, review articles, in vitro and animal studies, interventional studies, and duplicates), 11 articles underwent full-length review. 1 article was excluded because no outcome of interests reported and 2 articles were excluded because of absence of full text paper. Ultimately, our analysis included 8 observational studies (one case–control, five retrospective cohorts, and two prospective cohorts) involving 66,479 participants. Figure 1 illustrates the literature retrieval, review, and selection processes, while Table 1 presents the characteristics and quality assessment of the included studies.

### Characteristics and quality assessment

The majority of the included studies focused on a female population of 56%. All participants can classify into 2 groups which are 14,190 individuals with LVH and 52,289 individuals with non-LVH. Six studies utilized echocardiogram as the diagnostic tool for LVH detection, while two studies employed cardiac magnetic resonance imaging (MRI). In terms of AI classifier, neural network (NN) was used as an AI model in 6 studies which are convolutional NN (CNN) in 3 studies, ensemble NN (ENN) in 2 studies, and 1 study of back propagation NN (BPN) and non-NN was used in the other 2 studies which consist of Bayesian additive regression trees (BART) and C5.0 algorithm. The median QUADAS score of included studies was ranging from 12 to 13 which indicates high quality of included studies.

### Diagnostic accuracy of artificial intelligence for the presence of LVH (Fig. 2)

**Figure 2:**
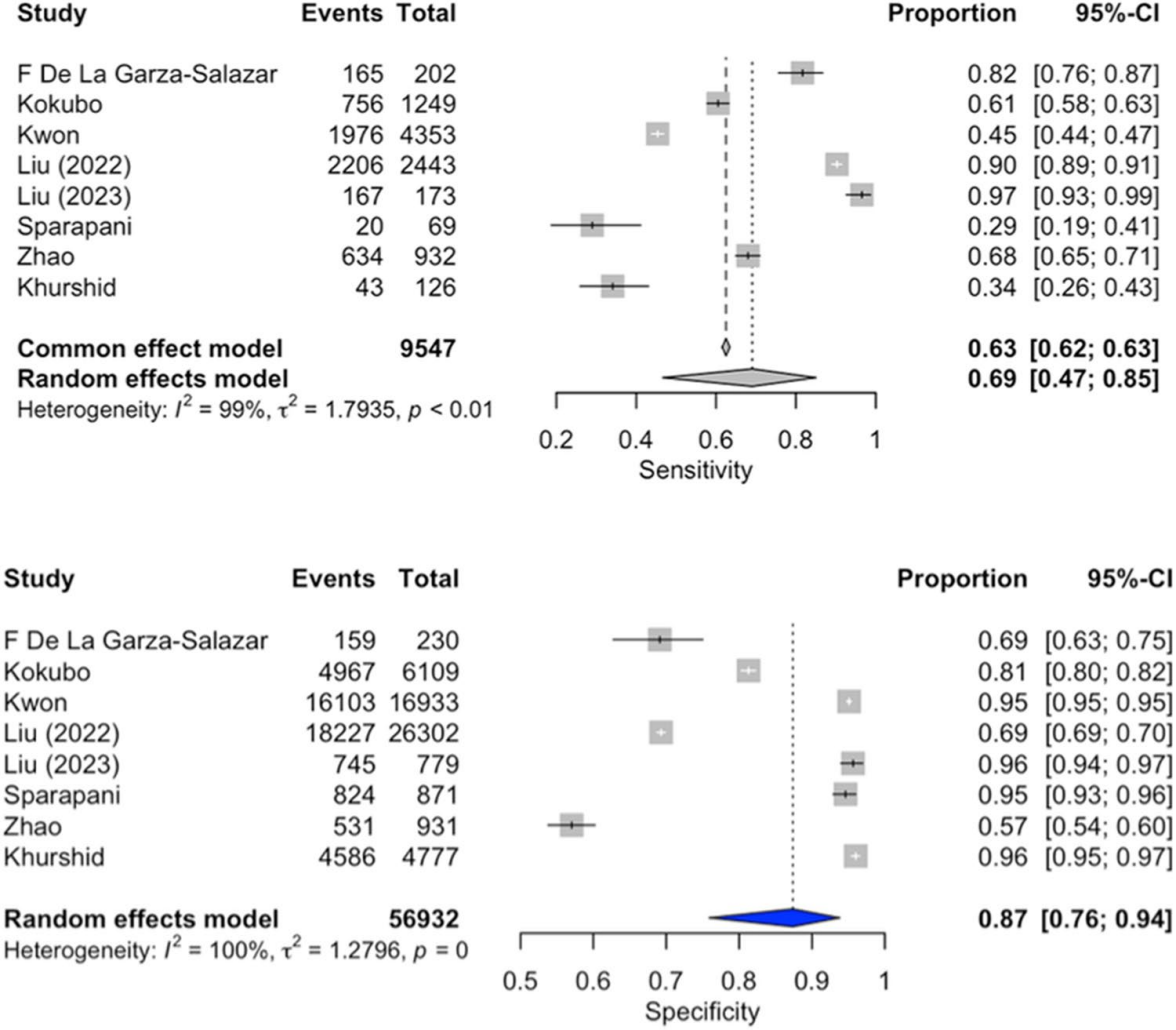
Forest plot of sensitivity and specificity of Artificial Intelligence for the presence of LVH.

**Figure 3:**
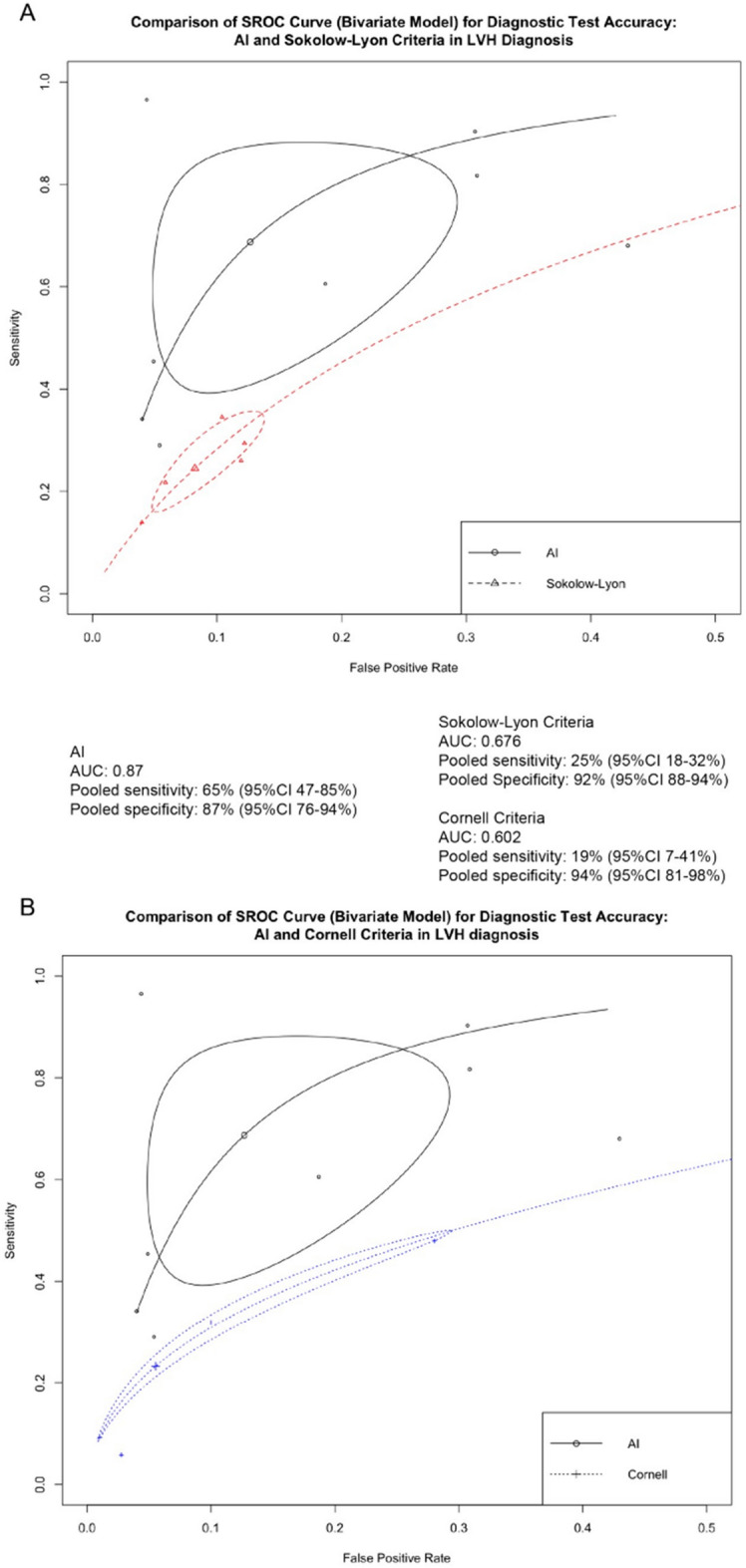
Summary receiver operating characteristic (SROC) of the diagnostic accuracy of artificial intelligence for the presence of LVH, compared with Sokolow–Lyon’s (**a**), and Cornell’s criteria (**b**).

The overall analysis^9–16^ revealed an area under the Summary Receiver Operating Characteristic (SROC) curve of 0.87 (Fig. 3). The pooled sensitivity was 69% (95% CI 47–85%), and the pooled specificity was 87% (95% CI 76–94%). Considerable heterogeneity was observed among the included studies (I^2^ = 100%).

### Diagnostic accuracy of Sokolow–Lyon’s criteria for the presence of LVH (Fig. 4)

**Figure 4:**
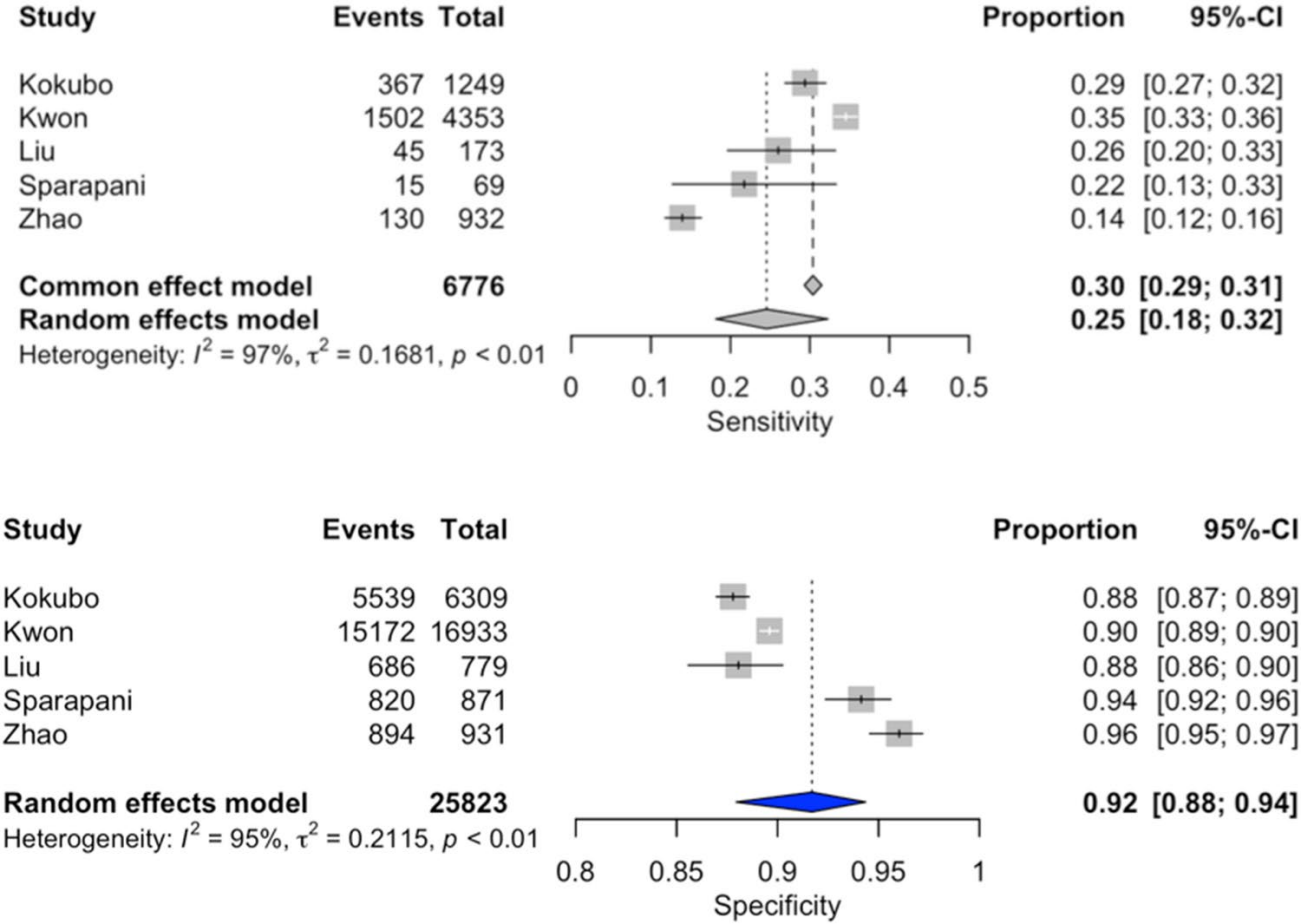
Forest plot of sensitivity and specificity of Sokolow–Lyon’s criteria for the presence of LVH.

The overall analysis^9–16^ yielded an area under the SROC curve of 0.68 (Fig. 3a). The pooled sensitivity was 25% (95% CI 18–32%), and the pooled specificity was 92% (95% CI 88–94%). Considerable heterogeneity was observed among the included studies (I^2^ = 95%).

### Diagnostic accuracy of Cornell’s criteria for the presence of LVH (Fig. 5)

**Figure 5:**
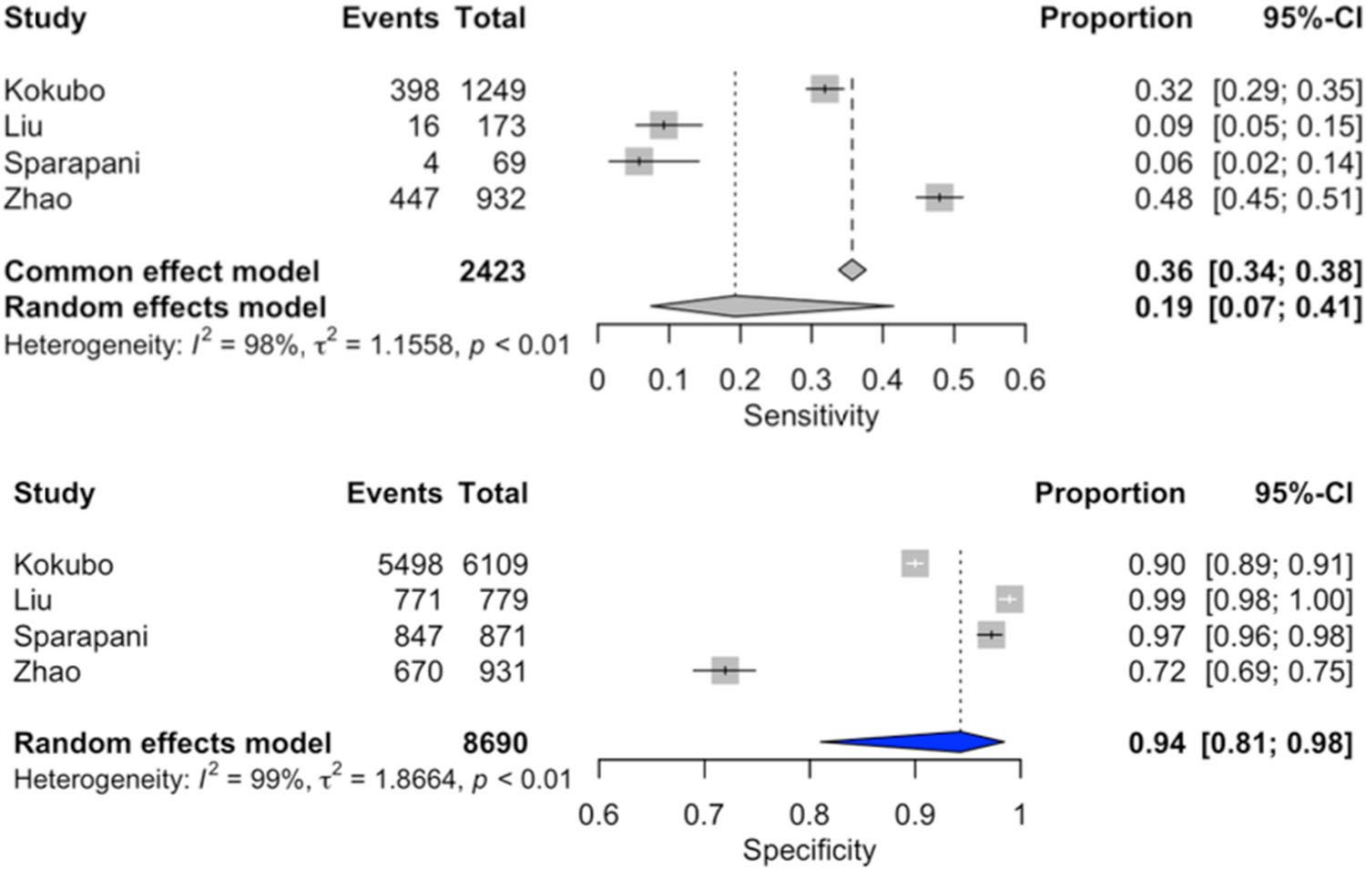
Forest plot of sensitivity and specificity of Cornell’s criteria for the presence of LVH.

The overall analysis^9–16^ yielded an area under the SROC curve of 0.60 (Fig. 3b). The pooled sensitivity was 19% (95% CI 7–41%), and the pooled specificity was 94% (95% CI 81–98%). Considerable heterogeneity was observed among the included studies (I^2^ = 98%).

### Publication bias

**Figure 6:**
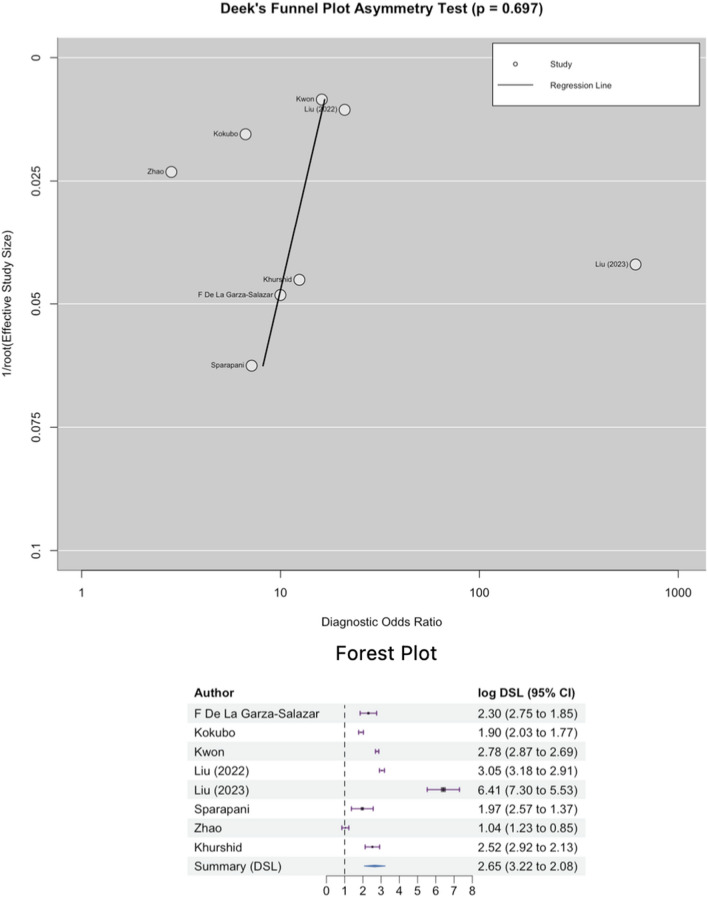
Deek’s funnel plot of publication bias.

The slope coefficient of Deek’s funnel plot exhibited a relatively symmetrical distribution, as depicted in Fig. 6, with a P-value of 0.9177. This finding implies the absence of publication bias.

## Discussion

Our study aimed to assess the diagnostic accuracy of AI in detecting LVH with electrocardiography and compare it to the conventional criteria, including Cornell’s and Sokolow–Lyon’s criteria. Our findings suggest that, by SROC, AI was associated with higher diagnostic accuracy as compared to the other two conventional criteria’s. Further, we observed a notable increase in sensitivity for LVH detection by AI, when compared to Sokolow–Lyon’s and Cornell’s criteria. However, the specificity of AI was comparatively lower than that of the conventional criteria. Due to its enhanced sensitivity, AI could be used as a screening tool in conjunction with conventional criteria to identify LVH.

To improve diagnostic performance in ECG detection of LVH, several ECG criteria have been iteratively refined over decades^20^. For instance, Peguero et al. proposed a novel ECG criterion that outperformed Cornell’s voltage criteria on sensitivity, 62% over 35%, respectively^19^. Conversely, the previous study focusing on patients over the age of 65 found Cornell’s Product criteria with improved performance, an AUC of 0.62, albeit yielding suboptimal results^21^. According to these pre-existing publications, the primary limitations of conventional criteria have been identified as a disparity between sensitivity and specificity, as well as the exclusion of ECG abnormalities that bear prognostic significance^3,22–24^. To address these limitations, machine learning and deep learning-based AI techniques have been employed, enabling the utilization of extensive ECG-LVH data and highly applicable ECG features. The ability of AI algorithms to incorporate diverse types of input data, including images and waveforms, has proven to be crucial. For example, Kwon et al. incorporated not only variables such as the presence of atrial fibrillation or flutter, QT interval, QTc, QRS duration, R-wave axis, and T-wave axis as input data but also raw ECG data in a two-dimensional numeric format^9^.

Our study incorporates several machine learning methods that have been previously developed and employed in relevant research. For instance, Sparapani et al.^13^ devised the BART-LVH criteria for detecting LVH by leveraging BART, a machine-learning technique. They utilized patient characteristics such as demographics, biometrics, and cardiovascular disease risk factors like blood pressure and body mass index. Additionally, De la Garza-Salazar et al.^11^ employed logistic regression for data dimensionality reduction and subsequently constructed a decision tree model using the C5.0 algorithm. This decision tree model incorporated multiple ECG measurements, including ST abnormalities, S wave voltage in lead V4, intrinsicoid deflection in lead V6 (qR duration ≥ 0.05 s), negative deflection of P wave in lead V1, and R wave voltage in lead aVR. This approach holds promise for real-life applications due to its simplicity and utilization of basic parameters measured by ECG machines.

Another successful example of a non-black box model in diagnosing echo-LVH, demonstrated by De la Garza-Salazar et al., is the Cardiac Hypertrophy Computer-based Model (CHCM). This AI model achieved balanced sensitivity and specificity, surpassing the accuracy of traditional criteria like Cornell and Sokolow–Lyon. By integrating diverse types of input data, including ECG quantitative data and patient characteristics, AI algorithms offer a promising avenue for improving LVH detection accuracy^25^.

The utilization of AI and black box models for diagnosing LVH holds promise for advancing ECG analyses. However, a notable drawback of AI and machine learning is their lack of transparency regarding the reasoning behind their diagnoses, potentially leading to the loss of prognostic markers. For instance, while the strain pattern in ECG is recognized as an important marker of LVH, it also serves as a prognostic indicator in various clinical conditions, as demonstrated in studies such as the Framingham Heart Study and numerous cohorts^26–28^.

To strike a balance between diagnostic accuracy and clinical significance, one approach involves harnessing non-black box AI models to extract and analyze a broader range of ECG parameters. By embracing interpretable AI techniques, researchers can uncover insights into the relationships between ECG features and the prognosis of LVH, thus ensuring a more comprehensive understanding of the diagnostic process and its implications for patient care.

### Study limitations

There are a few limitations in our meta-analysis. First, majorities of the included studies were observational. Therefore, residual confounders were not completely excluded, deleteriously complicating the results. The utilization of AI in diagnosing conditions may lead to both overestimation and underestimation of its accuracy. Second, the heterogeneity of this study was significant due to the inclusion of studies that featured various study designs including types of AI methods, demographic data, individuals’ underlying diseases, and other factors that could not be determined. Hence, the interpretation of this analysis must be cautiously utilized with the appropriate and applicable contexts. Lastly, our study did not aim to specifically assess the accuracy of the LVH detection algorithms. Instead, our primary objective was to offer an overview of the overall validity of the newly developed LVH using AI.

## Conclusion

To the best of our knowledge, this is the most extensive study to date utilizing large-scale observational studies to evaluate the diagnostic accuracy of AI. Our findings indicate that the use of AI in detecting LVH may help improve diagnostic performance compared to ECG. Nonetheless, given the limitations, further research is necessary to explore the clinical implications, generalizability, and cost–benefit of using AI for LVH diagnosis.

## Data Availability

All data generated or analysed during this study are included in this published article.
